# Echocardiographic parameters in French Bulldogs, Pugs and Boston Terriers with brachycephalic obstructive airways syndrome

**DOI:** 10.1186/s12917-023-03600-9

**Published:** 2023-02-15

**Authors:** M. Brložnik, A. Nemec Svete, V. Erjavec, A. Domanjko Petrič

**Affiliations:** University of Ljubljana, Veterinary Faculty, Small Animal Clinic, Gerbičeva 60, 1000 Ljubljana, Slovenia

**Keywords:** Dog, Brachycephaly, BOAS, Obstructive sleep apnea, Echocardiography, Right heart

## Abstract

**Background:**

In this prospective study, we hypothesized that dogs with signs of brachycephalic obstructive airway syndrome (BOAS) would show differences in left and right heart echocardiographic parameters compared with brachycephalic dogs without signs of BOAS and non-brachycephalic dogs.

**Results:**

We included 57 brachycephalic (30 French Bulldogs 15 Pugs, and 12 Boston Terriers) and 10 non-brachycephalic control dogs.

Brachycephalic dogs had significantly higher ratios of the left atrium to aorta and mitral early wave velocity to early diastolic septal annular velocity; smaller left ventricular (LV) diastolic internal diameter index; and lower tricuspid annular plane systolic excursion index, late diastolic annular velocity of the LV free wall, peak systolic septal annular velocity, late diastolic septal annular velocitiy, and right ventricular global strain than non-brachycephalic dogs.

French Bulldogs with signs of BOAS had a smaller diameter of the left atrium index and right ventricular systolic area index; higher caudal vena cava at inspiration index; and lower caudal vena cava collapsibility index, late diastolic annular velocity of the LV free wall, and peak systolic annular velocity of the interventricular septum than non-brachycephalic dogs.

**Conclusions:**

The differences in echocardiographic parameters between brachycephalic and non-brachycephalic dogs, brachycephalic dogs with signs of BOAS and non-brachycephalic dogs, and brachycephalic dogs with and without signs of BOAS indicate higher right heart diastolic pressures affecting right heart function in brachycephalic dogs and those with signs of BOAS. Most changes in cardiac morphology and function can be attributed to anatomic changes in brachycephalic dogs alone and not to the symptomatic stage.

## Background

Brachycephalic dogs have a shortened skull with compressed nasal passages and abnormal pharyngeal anatomy, resulting in upper airway obstruction [1, 2]. Brachycephalic Obstructive Airway Syndrome (BOAS) is a complex syndrome characterized by numerous primary airway abnormalities: stenotic nares, abnormal conchal growth, elongated soft palate, and hypoplastic trachea [1, 3, 4]. These primary components of BOAS lead to increased respiratory effort and create negative pressure within the airways, resulting in additional secondary abnormalities: everted laryngeal saccules, laryngeal collapse, everted tonsils, and edematous pharyngeal tissue [2–5]. Clinical signs include stertor, stridor, snoring, restlesness at night, retching, gagging, regurgitation, vomiting, exercise and heat intolerance at rest and during daily activities, including running, prolonged recovery after exercise, syncope, and others [6–8]. The most common brachycephalic breeds in which BOAS occurs are French Bulldogs (FB), Pugs (P), Boston Terriers (BST) and English Bulldogs [1, 6, 9]. Despite the well-documented conformation-related health problems, the number of brachycephalic dogs is increasing [10–12]. Selection for extreme brachycephaly has resulted in more upper airway obstruction and dogs of a younger age severely affected by BOAS [10, 12, 13].

Brachycephalic dogs share many similarities with human patients affected by Obstructive Sleep Apnea (OSA) [14–19], a disordered sleep breathing condition defined by repetitive cessations of breathing due to periodic episodes of upper airway obstruction [19–22]. Various structural and functional changes of the heart have been noted in OSA patients, such as a marked increase in cardiac preload and afterload leading to atrial stretch, enlargement, remodeling, and fibrosis [20–26]. Obstructive sleep apnea is an independent risk factor for cardiovascular diseases such as ischemic heart disease, systemic hypertension, and heart failure [19, 27]. Similarly, increased upper airway resistance in brachycephalic dogs most likely affects their hearts as well. Severe cardiopulmonary changes, i.e. pulmonary hypertension and *cor pulmonale*, have been reported in children with chronic adeno-tonsillar hypertrophy and OSA, which are reversible with adenotonsillectomy [28–32].

In FB, a larger ratio of the left atrium to aorta in short axis (LA/Ao), decreased left ventricular internal diameter in systole (LVIDs), and higher fractional shortening (FS) were noted compared to control Beagle dogs [33]. In P, significant differences were observed in the left ventricular internal diameter in diastole (LVIDd), interventricular septum and posterior wall thickness in diastole (IVSd and LVPWd, respectively), and tricuspid annular plane systolic motion excursion (TAPSE) compared to interbreed reference values [34].

The aim of this study was to determine possible echocardiographic differences between brachycephalic and non-brachycephalic dogs and to evaluate possible echocardiographic changes due to BOAS. Our hypothesis was that brachycephalic dogs and dogs with signs of BOAS have a larger right heart due to respiratory problems, which may also lead to changes in left heart dimensions and bilateral flow characteristics, as well as to alterations in cardiac function.

## Results

We included 57 brachycephalic dogs: 30 FB, 15 P, and 12 BST. Eighteen brachycephalic (31.6%) dogs were without clinical signs of BOAS and 39 dogs (68.4%) had signs of BOAS (Table 1). All dogs with clinical signs of BOAS had difficulties breathing in the lateral recumbence on echocardiographic examination, which was not the case in dogs without signs of BOAS. Mild tricuspid regurgitation (TR) was noted in eight brachycephalic dogs (8/57, 14%) and in four brachycephalic dogs (4/57, 7%), maximal velocity of TR was consistent with mild pulmonary hypertension (31–50 mm Hg). Mild mitral regurgitation was noted in four brachycephalic dogs (4/57, 7%).

**Table 1:** Characteristics of 57 brachycephalic dogs

Group	French Bulldogs	Pugs	Boston Terriers	All brachycephalic dogs
All	30 (52.6%)	15 (26.3%)	12 (21.1%)	57 (100%)
Females	20 (66.7%)	6 (40%)	6 (50%)	32 (56.1%)
Males	10 (33.3%)	9 (60%)	6 (50%)	25 (43.9%)
Without signs of BOAS	10 (33.3%) 10 F	5 (33.3%) 3 F, 2 M	3 (25%) 3 F	18 (31.6%) 16 F, 2 M
With signs of BOAS	20 (66.7%) 10 F, 10 M	10 (66.7%) 3 F, 7 M	9 (75%) 3 F, 6 M	39 (68.4%) 16 F, 23 M
BOAS 1*	2 (10%) 2 F	0	1 (11.1%) 1 M	3 (7.7%) 2 F, 1 M
BOAS 2*	13 (65%) 6 F, 7 M	5 (50%) 1 F, 4 M	6 (66.7%) 2 F, 4 M	24 (61.5%) 9 F, 15 M
BOAS 3*	5 (25%) 2 F, 3 M	5 (50%) 2 F, 3 M	2 (22.2%) 1 F, 1 M	12 (30.8%) 5 F, 7 M

*BOAS* Brachycephalic obstructive airway syndrome, *F* Female, *M* Male

^*^The dogs were classified as grade 1 – 3 based on the decrease in laryngeal airway radius after the soft palate was retrieved rostrally in a position that mimics the situation after folded-flap palatoplasty [35]

Ten healthy non-brachycephalic dogs with normal cardiac auscultation, four females and six males (mixed dolichocephalic dogs (*n* = 8; 4M/4F), a Border Collie (*n* = 1; M), and a German Miniature Spitz (*n* = 1; M)), served as controls. No mitral nor tricuspid insufficiencies were noted in control dogs.

### All brachycephalic dogs compared to non-brachycephalic dogs

The age and weight differences between brachycephalic and non-brachycephalic dogs are shown in Table 2.

**Table 2:** Age, weight, and 2D and M-mode echocardiographic parameters in brachycephalic and non-brachycephalic dogs

**Variable** (unit)	**French Bulldogs (*n* = 30)**	**Pugs (*n* = 15)**	**Boston Terriers (*n* = 12)**	**Brachycephalic dogs (*n* = 57)**	**Non-brachycephalic dogs (*n* = 10)**
**Age** (years)	1.52 (1.06–2.57)**	3.08 (1.12–4.42)	3.22 (1.62–6.56)	2.22 (1.26–4.07)	3.48 (2.86–4.85)
**Weight** (kg)	10.0 (9.15–12.25)**	9.0 (7.70–10.0)**	7.15 (6.35–9.93)**	9.5 (8.0–11.6)**	13.3 (10.90–14.70)
**LA/Ao**	1.56 (1.44–1.60)**	1.49 (1.45–1.62)	1.55 (1.50–1.68)**	1.55 (1.47–1.62)**	1.44 (1.35–1.54)
**AoI** (cm/kg^1/3^)	0.71 (0.65–0.75)	0.67 (0.62–0.77)	0.73 (0.63–0.76)	0.71 (0.64–0.76)	0.75 (0.71–1.18)
**LASaxI** (cm/kg^1/3^)	1.10 (1.01–1.15)	1.01 (0.94–1.12)	1.09 (1.03–1.13)	1.08 (1.01–1.14)	1.06 (1.01–1.19)
**LALaxI** (cm/kg^1/3^)	1.16 (1.07–1.20)	1.0 (0.97–1.12)**	1.24 (1.20–1.30)	1.16 (1.03–1.22)	1.20 (1.13–1.25)
**IVSdI** (cm/kg^1/3^)	0.33 (0.28–0.37)	0.34 (0.29–0.35)	0.39 (0.32–0.44)	0.34 (0.29–0.38)	0.36 (0.33–0.40)
**LVIDdI** (cm/kg^1/3^)	1.35 (1.27–1.48)	1.27 (1.15–1.34)**	1.45 (1.39–1.63)	1.35 (1.27–1.47)**	1.48 (1.30–1.56)
**LVPWdI** (cm/kg^1/3^)	0.35 (0.31–0.39)	0.34 (0.29–0.37)	0.36 (0.35–0.40)	0.35 (0.31–0.38)	0.33 (0.31–0.39)
**IVSsI** (cm/kg^1/3^)	0.43 (0.38–0.48)**	0.39 (0.36–0.43)**	0.57 (0.44–0.60)	0.43 (0.38–0.53)	0.51 (0.42–0.62)
**LVIDsI** (cm/kg^1/3^)	0.89 (0.75–0.98)	0.91 (0.82–0.93)	0.85 (0.75–0.98)	0.89 (0.77–0.97)	1.01 (0.88–1.03)
**LVPWsI** (cm/kg^1/3^)	0.46 (0.42–0.54)	0.43 (0.38–0.48)**	0.54 (0.51–0.58)**	0.48 (0.42–0.54)	0.49 (0.46–0.52)
**FS** (%)	36.5 (31.0–43.0)	31.0 (25.0–37.0)	40.0 (33.75–47.75)**	36.0 (31.0–43.0)	33.0 (28.75–36.75)
**RVIDbasI** (cm/kg^1/3^)	0.70 (0.60–0.77)	0.65 (0.57–0.73)**	0.73 (0.63–0.78)	0.70 (0.60–0.76)	0.68 (0.54–0.76)
**RVIDmidI** (cm/kg^1/3^)	0.72 (0.65–0.77)	0.63 (0.61–0.77)	0.83 (0.64–0.86)	0.71 (0.63–0.82)	0.72 (0.65–0.85)
**RVIDlongI**(cm/kg1/3)	1.30 (1.18–1.39)	1.25 (1.19–1.36)	1.32 (1.13–1.45)	1.30 (1.18–1.39)	1.27 (1.15–1.38)
**RVIDlong/RVIDmid**	1.78 (1.59–2.06)	1.93 (1.65–2.14)	1.75 (1.56–1.90)	1.83 (1.60–2.06)	1.79 (1.57–2.04)
**RVAdI** (cm^2^/kg^2/3^)	0.77 (0.70–0.89)	0.57 (0.50–0.64)**	0.88 (0.61–1.01)	0.75 (0.60–0.90)	0.85 (0.72–1.02)
**RVAsI** (cm^2^/kg^2/3^)	0.40 (0.35–0.45)	0.27 (0.23–0.39)**	0.44 (0.31–0.56)	0.39 (0.31–0.48)	0.52 (0.39–0.58)
**FAC** (%)	47.5 (43.0–56.0)	47.0 (34.0–53.0)	49.50 (39.0–55.5)	48.0 (41.0–54.0)	45.50 (39.75–46.25)
**RAAI** (cm^2^/kg^2/3^)	0.73 (0.66–0.86)	0.60 (0.48–0.80)	0.67 (0.55–0.85)	0.71 (0.60–0.85)	0.76 (0.66–4.75)
**TAPSEI** (cm/kg^1/3^)	0.50 (0.42–0.57)**	0.47 (0.35–0.56)**	0.52 (0.41–0.63)	0.49 (0.41–0.58)**	0.62 (0.55–0.67)
**MAPSEI** (cm/kg^1/3^)	0.39 (0.34–0.48)	0.37 (0.29–0.40)**	0.47 (0.41–0.54)	0.40 (0.33–0.47)	0.44 (0.39–0.52)
**CVCII** (cm/kg^1/3^)	0.23 (0.17–0.28)	0.16 (0.13–0.22)	0.25 (0.22–0.32)**	0.23 (0.16–0.28)	0.20 (0.20–0.23)
**CVCEI** (cm/kg^1/3^)	0.39 (0.35–0.45)	0.35 (0.29–0.42)	0.43 (0.38–0.48)**	0.40 (0.35–0.44)	0.39 (0.35–0.44)
**CVCCI** (%)	37.5 (33.3–50.0)**	50.0 (43.7–53.6)	40.0 (37.5–42.9)**	42.86 (34.1–50.0)	50 (44.1–51.79)

In brachycephalic groups, both dogs with and without signs of brachycephalic obstructive airway syndrome are included, data are presented as medians (interquartile range: 25th – 75th percentile), *AoI* Aortic diameter index, *CVCEI* Caudal vena cava at expiration index, *CVCII* Caudal vena cava at inspiration index, *CVCCI* Caudal vena cava collapsibility index = (CVCE–CVCI)/CVCE*100%, *FAC* Fractional area change of right ventricle, *FS* Fractional shortening of left ventricle, *I* Weight dependable variable index = one dimensional as variable/weight^1/3^ and two dimensional as variable/weight^2/3^, *IVSdI* Thickness of interventricular septum in diastole index, *IVSsI* Thickness of interventricular septum in systole index, *LALaxI* Diameter left atrium in long axis index, *LASaxI* Diameter of left atrium in short axis index, *LA/Ao* Left atrium in short axis to aortic diameter ratio, *LVIDdI* Left ventricular internal diameter in diastole index, *LVIDsI* Left ventricular internal diameter in systole index, *LVPWdI* Thickness of left ventricular posterior wall in diastole index, *LVPWsI* Thickness of left ventricular posterior wall in systole index, *MAPSEI* Mitral annular plane systolic excursion index, *RAAI* Right atrial area index, *RVAdI* Right ventricular area in diastole index, *RVAsI* Right ventricular area in systole index, *RVIDbasI* Right ventricular internal diameter at base index, *RVIDmidI* Right ventricular internal diameter in mid cavity index, *RVIDlongI* Right ventricular longitudinal diameter index, *TAPSEI* Tricuspid annular plane systolic excursion index

^**^ = significant differences to non-brachycephalic dogs

#### Brachycephalic dogs (with and without signs of BOAS) compared to non-brachycephalic dogs

Compared to non-brachycephalic dogs, brachycephalic dogs exhibited a larger LA/Ao ratio (*p* = 0.019); higher mitral early wave velocity to early diastolic septal annular velocity (MVE/Ei) ratio (*p* = 0.049); smaller left ventricular internal diameter in diastole index (LVIDdI) (*p* = 0.037); lower tricuspid annular plane systolic excursion index (TAPSEI) (*p* = 0.005), late diastolic annular velocity of the left ventricular free wall (Am) (*p* = 0.003), peak systolic septal annular velocity (Si) (*p* = 0.003), and late diastolic septal annular velocity (Ai) (*p* = 0.049); and decreased right ventricular global strain GSRV (*p* = 0.022) (Tables 2 and 3).

**Table 3:** Doppler-derived echocardiographic parameters in brachycephalic and non-brachycephalic dogs

**Variable** (unit)	**French Bulldogs (*n* = 30)**	**Pugs (*n* = 15)**	**Boston Terriers (*n* = 12)**	**Brachycephalic dogs (*n* = 57)**	**Non-brachycephalic dogs (*n* = 10)**
**MVE** (m/s)	0.79 (0.69–0.91)**	0.67 (0.57–0.74)	0.74 (0.62–0.84)	0.74 (0.67–0.83)	0.72 (0.60–0.79)
**MVA** (m/s)	0.54 (0.50–0.65)	0.45 (0.42–0.52)**	0.49 (0.39–0.57)	0.52 (0.44–0.60)	0.53 (0.46–0.61)
**MVE/A**	1.43 (1.26–1.62)	1.5 (1.17–1.62)	1.62 (1.13–1.79)	1.50 (1.22–1.67)	1.38 (1.19–1.56)
**TVE** (m/s)	0.68 (0.57–0.81)	0.62 (0.45–0.72)	0.69 (0.55–0.77)	0.65 (0.55–0.78)	0.65 (0.50–0.76)
**TVA** (m/s)	0.44 (0.37–0.56)	0.38 (0.29–0.46)	0.39 (0.37–0.48)	0.40 (0.36–0.50)	0.47 (0.36–0.52)
**TVE/A**	1.51 (1.34–1.65)	1.69 (1.45–1.90)	1.61 (1.42–1.77)	1.52 (1.39–1.72)	1.45 (1.27–1.79)
**MVE/TVE**	1.13 (1.01–1.55)	1.10 (0.95–1.38)	1.09 (0.92–1.42)	1.12 (0.99–1.44)	1.13 (0.97–1.25)
**MVA/TVA**	1.19 (1.04–1.47)	1.31 (0.90–1.55)	1.19 (1.0–1.38)	1.20 (1.01–1.46)	1.05 (0.95–1.33)
**Sm** (cm/s)	10.60 (7.35–12.35)	6.40 (5.40–9.10)**	9.60 (7.05–10.83)	8.65 (6.4–10.98)	8.40 (7.7–12.15)
**Em** (cm/s)	11.10 (8.70–12.9)	7.50 (6.10–9.00)**	8.75 (7.0–11.05)	9.15 (7.15–11.88)	10.50 (8.3–12.15)
**MVE/Em** (cm/s)	7.50 (6.35–8.75)	8.20 (7.30–11.00)**	8.35 (6.22–9.8)	7.65 (6.6–9.48)	6.70 (5.7–9.05)
**Am** (cm/s)	6.00 (4.60–7.80)**	5.30 (3.60–6.90)**	7.75 (6.83–8.65)	6.50 (4.50–8.20)**	9.80 (7.85–10.80)
**Si** (cm/s)	6.70 (5.05–9.55)**	6.40 (5.20–6.90)**	7.95 (5.3–8.47)**	6.60 (5.13–8.65)**	9.40 (8.0–13.35)
**Ei** (cm/s)	6.90 (5.45–8.65)	5.80 (4.80–7.10)**	7.00 (4.65–7.85)	6.60 (4.11–7.98)	8.10 (6.45–9.10)
**MVE/Ei** (cm/s)	11.20 (9.75–13.95)	10.50 (8.90–13.80)	10.80 (9.95–27.35)	11.0 (9.43–13.80)**	9.70 (7.10–11.30)
**Ai** (cm/s)	5.60 (4.50–7.40)	5.10 (4.40–7.40)	5.40 (4.58–8.50)	5.40 (4.43–7.38)**	7.20 (5.75–8.85)
**St** (cm/s)	8.90 (7.30–13.0)	7.90 (6.50–11.30)	11.10 (7.23–11.58)	8.90 (7.2–11.45)	9.40 (8.15–18.80)
**Et** (cm/s)	9.10 (8.35–11.45)	8.80 (7.40–10.90)	10.30 (9.08–11.65)	9.60 (8.3–11.35)	10.10 (6.60–12.10)
**TVE/Et** (cm/s)	6.86 (5.62–7.70)	6.59 (5.04–8.32)**	7.16 (4.61–7.52)	6.87 (5.25–7.79)	7.23 (5.90–8.45)
**At** (cm/s)	8.60 (7.10–11.55)	6.90 (5.10–8.4)	8.65 (6.50–9.98)	7.70 (6.4–10.0)	9.90 (7.55–15.45)
**GSAplax** (%)	19.20 (15.0–22.0)	17.95 (13.86–20.9)	20.2 (15.8–24.6)	19.15 (15.33–22.30)	18.60 (16.35–19.93)
**GSLV4ch** (%)	19.80 (16.6–20.9)	16.95 (13.4–20.45)	19.8 (17.1–21.8)	19.25 (16.25–20.83)	18.2 (17.25–18.95)
**GSLV2ch** (%)	19.60 (16.25–22.8)	16.90 (7.96–18.7)	20.4 (14.8–27.2)	18.20 (14.75–22.35)	19.55 (16.50–22.33)
**GSLV** (%)	19.33 (17.25–21.75)	17.37 (13.07–20.43)**	21.7 (16.1–23.5)	19.30 (16.1–21.75)	19.0 (17.38–20.73)
**GSRV** (%)	18.90 (14.6–23.41)**	16.90 (14.4–21.2)	21.5 (19.1–25.35)	18.70 (14.80–22.85)**	22.7 (20.4–26.8)

In brachycephalic groups, both dogs with and without signs of brachycephalic obstructive airway syndrome are included, data are presented as medians (interquartile range: 25th – 75th percentile), *Ai* Late diastolic interventricular annular velocity, *Am* Late diastolic mitral annular velocity, *At* Late diastolic tricuspid annular velocity, *Ei* Early diastolic interventricular annular velocity, *Em* Early diastolic mitral annular velocity, *Et* Early diastolic tricuspid annular velocity, *GSAplax* Left ventricular global strain in apical long axis 5 chamber view, *GSLV* Mean left ventricular global strain, *GSLV4ch* Left ventricular global strain in apical long axis 4 chamber view, *GSLV2ch* Left ventricular global strain in apical long axis 2 chamber view, *GSRV* Right ventricular global strain in apical long axis view, *MVA* Late diastolic mitral wave, *MVE* Early diastolic mitral wave, *MVE/A* Early diastolic mitral wave to late diastolic mitral wave ratio, *Si* Peak systolic interventricular annular velocity, *Sm* Peak systolic mitral annular velocity, *St* Peak systolic tricuspid annular velocity, *TVA* Late diastolic tricuspid wave, *TVE* Early diastolic tricuspid wave, *TVE/A* Early diastolic tricuspid wave to late diastolic tricuspid wave ratio

^**^ = significant differences to non-brachycephalic dogs

#### All French Bulldogs (with and without signs of BOAS) compared to non-brachycephalic dogs

Compared to non-brachycephalic dogs, FB had a larger LA/Ao ratio (*p* = 0.033); higher mitral early wave velocity (MVE) (*p* = 0.047); smaller thickness of the interventricular septum in systole index (IVSsI) (*p* = 0.032); and lower TAPSEI (*p* = 0.008), caudal vena cava collapsibility index (CVCCI) (*p* = 0.033), Si (*p* = 0.018), Am (*p* = 0.04), and GSRV (*p* = 0.027) compared to non-brachycephalic dogs (Tables 2 and 3).

#### All Pugs (with and without signs of BOAS) compared to non-brachycephalic dogs

Compared to non-brachycephalic dogs, P had a smaller diameter of the left atrium in long axis index (LALaxI) (*p* = 0.003) and lower LVIDdI (*p* = 0.015), IVSsI (*p* = 0.015), thickness of the left ventricular posterior wall in systole index (LVPWsI) (*p* = 0.040), right ventricular area in diastole index (RVAdI) (*p* = 0.015), right ventricular area in systole index (RVAsI) (*p* = 0.027), TAPSEI (*p* = 0.007), mitral annular plane systolic excursion index (MAPSEI) (*p* = 0.008), mitral late diastolic wave velocity (MVA) (*p* = 0.045), peak systolic annular velocity of the left ventricular free wall (Sm) (*p* = 0.017), early diastolic annular velocity of the left ventricular free wall (Em) (*p* = 0.014), Am (*p* = 0.005), Si (*p* = 0.001), early diastolic annular velocity of the interventricular septum (Ei) (*p* = 0.008), and early diastolic tricuspid wave velocity to early diastolic annular velocity of the right ventricular free wall ratio (TVE/Et) (*p* = 0.005). Pugs had a higher early diastolic mitral wave velocity to early diastolic annular velocity of the left ventricular free wall ratio (MVE/Em) (*p* = 0.027) and lower mean left ventricular global strain (GSLV) (*p* = 0.05) compared to non-brachycephalic dogs (Tables 2 and 3).

#### All Boston terriers (with and without signs of BOAS) compared to non-brachycephalic dogs

Boston Terriers had larger LA/Ao (*p* = 0.016) and LVPWsI (*p* = 0.030), higher fractional shortening of the left ventricle (FS) (*p* = 0.016), higher caudal vena cava at inspiration index (CVCII) (*p* < 0.001) and caudal vena cava at expiration index (CVCEI) (*p* = 0.006). In addition, BST had lower CVCCI (*p* = 0.002) and Si (*p* = 0.023) compared to non-brachycephalic dogs (Tables 2 and 3).

### Brachycephalic dogs with signs of BOAS compared to non-brachycephalic dogs

The age and weight differences between brachycephalic dogs with signs of BOAS and non-brachycephalic dogs are shown in Table 4.

**Table 4:** Age, weight and 2D and M-mode echocardiographic parameters in brachycephalic breeds with and without signs of BOAS

**Variable** (unit)	**FB with signs of BOAS (*n* = 20)**	**FB without signs of BOAS (*n* = 10)**	**Pugs with signs of BOAS (*n* = 10)**	**Pugs without signs of BOAS (*n* = 5)**	**BST with signs of BOAS (*n* = 9)**
**Age** (years)	1.48 (1.14–2.60)✩	1.85 (0.71–2.90)	2.81 (0.98–6.51)	3.98 (2.11–8.16)	2.69 (1.53–2.69)
**Weight** (kg)	10.25 (9.28–12.75)✩	10.0 (8.9–12.03)	9.0 (6.90–10.28)✩	8.70 (7.75–10.60)	6.70 (6.10–10.70)✩
**LA/Ao**	1.56 (1.41–1.6)	1.55 (1.49–1.62)	1.49 (1.39–1.62)	1.56 (1.48–1.67)	1.51 (1.49–1.64)
**AoI** (cm/kg^1/3^)	0.73 (0.70–0.76)	0.68 (0.63–0.73)	0.74 (0.61–0.78)	0.64 (0.61–0.70)	0.75 (0.66–0.76)
**LASaxI** (cm/kg^1/3^)	1.10 (1.01–1.15)	1.10 (1.00–1.17)	1.02 (0.93–1.17)	1.01 (0.94–1.07)	1.06 (1.02–1.18)
**LALaxI** (cm/kg^1/3^)	1.10 (1.04–1.19)✩**	1.20 (1.15–1.24)**	0.99 (0.97–1.11)✩	1.01 (0.98–1.24)	1.27 (1.20–1.32)
**IVSdI** (cm/kg^1/3^)	0.33 (0.29–0.38)	0.32 (0.28–0.36)	0.34 (0.32–0.37)	0.29 (0.26–0.32)	0.40 (0.33–0.46)
**LVIDdI** (cm/kg^1/3^)	1.37 (1.26–1.52)	1.34 (1.31–1.50)	1.23 (1.15–1.35)✩	1.27 (1.19–1.32)	1.50 (1.39–1.61)
**LVPWdI** (cm/kg^1/3^)	0.35 (0.33–0.41)	0.34 (0.28–0.37)	0.34 (0.28–0.37)	0.33 (0.27–0.35)	0.36 (0.34–0.38)
**IVSsI** (cm/kg^1/3^)	0.45 (0.42–0.50)	0.39 (0.36–0.45)**	0.43 (0.36–0.54)	0.38 (0.33–0.39)	0.58 (0.45–0.63)
**LVIDsI** (cm/kg^1/3^)	0.87 (0.74–0.98)	0.92 (0.87–0.98)	0.89 (0.81–0.97)	0.91 (0.81–0.94)	0.83 (0.76–0.96)
**LVPWsI** (cm/kg^1/3^)	0.48 (0.42–0.57)	0.44 (0.41–0.48)	0.45 (0.41–0.48)	0.38 (0.35–0.47)	0.54 (0.50–0.58)
**FS** (%)	39.5 (34.25–43.0)✩	34.0 (28.25–38.50)	31.5 (22.7–39.0)	30.0 (27.0–42.5)	41.0 (36.50–50.0)✩
**RVIDbasI** (cm/kg^1/3^)	0.67 (0.60–0.75)	0.75 (0.60–0.84)	0.66 (0.51–0.74)	0.63 (0.59–0.70)	0.72 (0.64–0.78)
**RVIDmidI** (cm/kg^1/3^)	0.75 (0.67–0.79)	0.69 (0.61–0.79)	0.64 (0.61–0.81)	0.62 (0.61–0.75)	0.81 (0.63–0.86)
**RVIDlongI** (cm/kg1/3)	1.23 (1.18–1.37)	1.33 (1.17–1.45)	1.25 (1.20–1.36)	1.31 (1.18–0.69)	1.33 (1.21–1.47)
**RVIDlong/RVIDmid**	1.74 (1.56–1.90)	2.06 (1.59–2.13)	1.90 (1.62–2.18)	2.0 (1.92–2.14)	1.80 (1.63–1.94)
**RVAdI** (cm^2^/kg^2/3^)	0.76 (0.69–0.88)	0.78 (0.71–0.95)	0.56 (0.46–0.67)✩	0.57 (0.52–0.81)	0.90 (0.61–1.05)
**RVAsI** (cm^2^/kg^2/3^)	0.39 (0.35–0.44)✩	0.43 (0.31–0.53)	0.27 (0.23–0.43)✩	0.29 (0.25–0.53)	0.42 (0.34–0.55)
**FAC** (%)	49.5 (43.0–53.0)	46.5 (43.3–58.8)	47.0 (33.3–54.5)	47.0 (36.50–51.5)	50.0 (39.0–55.0)
**RAAI** (cm^2^/kg^2/3^)	0.74 (0.66–0.86)	0.73 (0.61–0.87)	0.70 (0.57–0.85)	0.58 (0.47–0.62)	0.71 (0.57–0.85)
**TAPSEI** (cm/kg^1/3^)	0.52 (0.44–0.58)	0.47 (0.40–0.54)	0.51 (0.34–0.60)**	0.35 (0.27–0.39)**	0.58 (0.41–0.64)
**MAPSEI** (cm/kg^1/3^)	0.41 (0.36–0.48)	0.37 (0.33–0.52)	0.36 (0.27–0.41)✩	0.38 (0.30–0.40)	0.51 (0.41–0.56)
**CVCII** (cm/kg^1/3^)	0.25 (0.18–0.30)✩	0.21 (0.14–0.27)	0.17 (0.14–0.23)	0.15 (0.12–0.19)	0.27 (0.23–0.32)✩
**CVCEI** (cm/kg^1/3^)	0.40 (0.35–0.49)	0.38 (0.33–0.43)	0.35 (0.28–0.43)	0.35 (0.27–0.35)	0.43 (0.57–0.85)✩
**CVCCI** (%)	34.8 (32.50–47.50)✩	41.43 (36.46–55.0)	47.73 (43.25–50.0)	50.0 (43.7–57.14)	34.85 (34.37–42.56)✩

Data are presented as medians (interquartile range: 25th – 75th percentile), *AoI* Aortic diameter index, *BOAS* Brachycephalic obstructive airway syndrome, *CVCEI* Caudal vena cava at expiration index, *CVCII* Caudal vena cava at inspiration index, *CVCCI* Caudal vena cava collapsibility index = (CVCE–CVCI)/CVCE*100%, *FAC* Fractional area change of right ventricle, *FS* Fractional shortening of left ventricle, *I* Weight dependable variable index = one dimensional as variable/weight^1/3^ and two dimensional as variable/weight^2/3^, *IVSdI* Thickness of interventricular septum in diastole index, *IVSsI* Thickness of interventricular septum in systole index, *LALaxI* Diameter left atrium in long axis index, *LASaxI* Diameter of left atrium in short axis index, *LA/Ao* Left atrium in short axis to aortic diameter ratio, *LVIDdI* Left ventricular internal diameter in diastole index, *LVIDsI* Left ventricular internal diameter in systole index, *LVPWdI* Thickness of left ventricular posterior wall in diastole index, *LVPWsI* Thickness of left ventricular posterior wall in systole index, *MAPSEI* Mitral annular plane systolic excursion index, *RAAI* Right atrial area index, *RVAdI* Right ventricular area in diastole index, *RVAsI* Right ventricular area in systole index, *RVIDbasI* Right ventricular internal diameter at base index, *RVIDmidI* Right ventricular internal diameter in mid cavity index, *RVIDlongI* Right ventricular longitudinal diameter index, *TAPSEI* Tricuspid annular plane systolic excursion index

✩ = significant differences between brachycephalic dogs with signs of BOAS and non-brachycephalic dogs (data presented in Tables 2 and 3), ** = significant differences between brachycephalic dogs with and without signs of BOAS

#### French Bulldogs with signs of BOAS compared to non-brachycephalic dogs

**Fig. 1:**
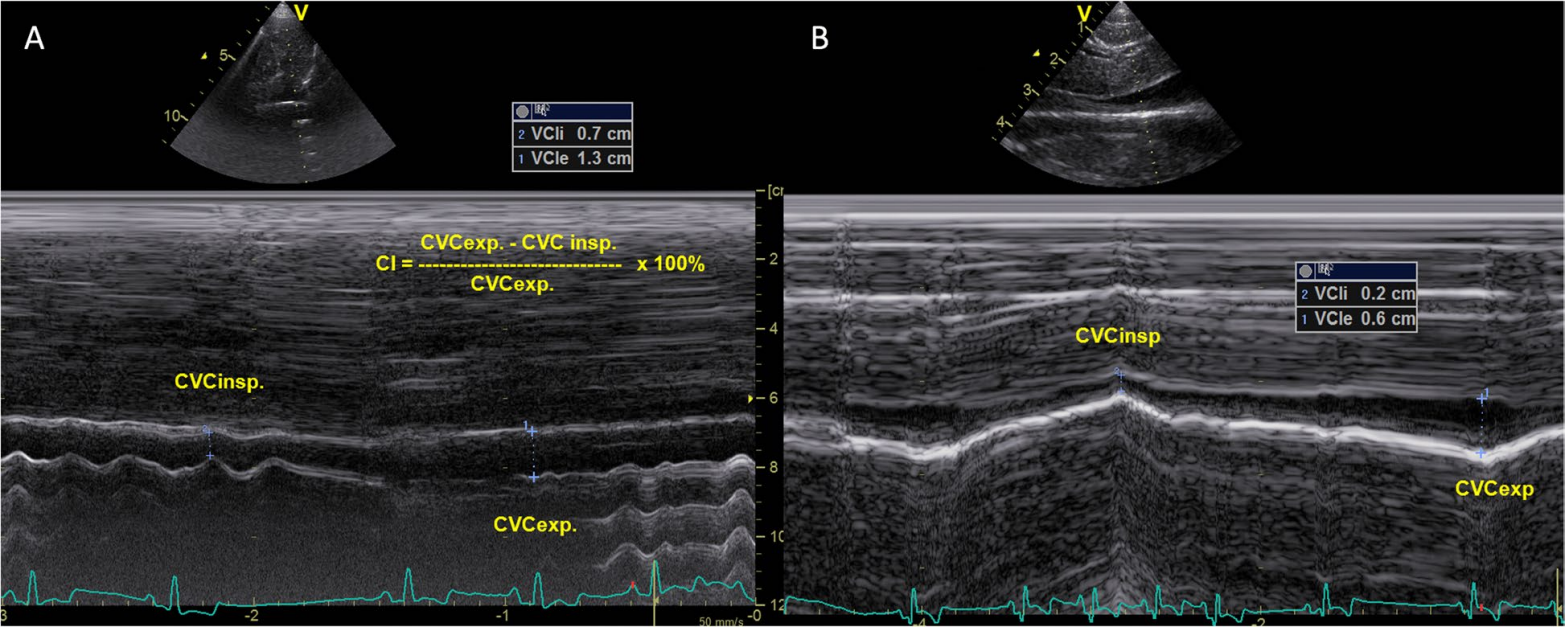
Caudal vena cava collapsibility index (CVCCI) was lower in FB with signs of BOAS (**A**) than in non-brachycephalic dogs (**B**). LEGEND: CVC insp. = caudal vena cava during inspiration, CVC exp. = caudal vena cava during expiration

French Bulldogs with signs of BOAS had smaller LALaxI (*p* = 0.016) and RVAsI (*p* = 0.028), higher FS (*p* = 0.019), higher CVCII (*p* = 0.047), and lower CVCCI (*p* = 0.018), Am (*p* = 0.005), and Si (*p* = 0.021) than dogs in the non-brachycephalic group (Tables 4 and 5). Figure 1 shows the visual difference between the lower CVCCI of the FB with signs of BOAS and the higher CVCCI of the non-brachycephalic dogs.

**Table 5:** Doppler-derived echocardiographic parameters in brachycephalic breeds with and without signs of BOAS

**Variable** (unit)	**FB with signs of BOAS (*n* = 20)**	**FB without signs of BOAS (*n* = 10)**	**Pugs with signs of BOAS (*n* = 10)**	**Pugs without signs of BOAS (*n* = 5)**	**BST with signs of BOAS (*n* = 9)**
**MVE** (m/s)	0.79 (0.68–0.96)	0.80 (0.71–0.98)	0.66 (0.56–0.76)	0.67 (0.56–0.75)	0.74 (0.64–0.84)
**MVA** (m/s)	0.58 (0.51–0.81)	0.53 (0.45–0.62)	0.45 (0.42–0.52)	0.42 (0.36–0.49)	0.51 (0.44–0.57)
**MVE/A**	1.37 (1.17–1.80)	1.50 (1.41–1.76)	1.45 (1.20–1.80)	1.52 (0.93–1.61)	1.66 (1.14–1.81)
**TVE** (m/s)	0.78 (0.60–0.87)**	0.57 (0.43–0.68)**	0.63 (0.50–0.75)	0.49 (0.34–0.70)	0.73 (0.57–0.82)
**TVA** (m/s)	0.47 (0.39–0.61)	0.39 (0.34–0.46)	0.39 (0.29–0.47)	0.31 (0.23–0.47)	0.41 (0.37–0.47)
**TVE/A**	1.52 (1.46–1.69)	1.36 (1.21–1.49)	1.89 (1.49–1.96)	1.60 (1.34–1.69)	1.62 (1.44–1.85)
**MVE/TVE**	1.04 (0.95–1.27)**	1.60 (1.34–1.73)**	1.05 (0.95–1.28)	1.37 (0.85–2.15)	1.04 (0.83–1.34)
**MVA/TVA**	1.16 (1.01–1.37)	1.36 (1.07–1.61)	1.22 (0.90–1.48)	1.45 (0.83–2.00)	1.16 (0.90–1.35)
**Sm** (cm/s)	11.0 (6.80–12.60)	8.85 (7.53–12.38)	7.0 (5.63–9.30)	6.30 (5.35–8.15)	10.20 (8.15–10.90)
**Em** (cm/s)	10.30 (8.20–12.90)	12.20 (9.65–13.20)	7.75 (6.88–9.93)	6.10 (4.35–8.75)	9.50 (7.80–13.0)
**MVE/Em** (cm/s)	7.60 (6.10–9.90)	7.10 (6.55–7.75)	7.80 (7.10–9.03)**	11.0 (8.15–13.75)**	7.10 (5.95–9.0)
**Am** (cm/s)	6.0 (4.50–8.10)✩	6.95 (4.78–7.90)	4.10 (2.80–7.68)✩	6.0 (4.90–7.85)	8.20 (6.85–9.65)
**Si** (cm/s)	5.90 (4.60–9.50)✩	7.50 (5.33–9.75)	6.65 (5.20–7.55)✩	5.30 (4.35–6.05)	8.10 (6.15–8.45)✩
**Ei** (cm/s)	7.50 (5.0–8.80)	6.60 (5.78–7.68)	6.50 (4.58–8.13)	5.50 (4.55–6.10)	7.20 (5.0–8.10)
**MVE/Ei** (cm/s)	10.70 (9.50–12.70)	12.30 (10.10–15.85)	10.35 (8.0–14.08)	11.80 (9.75–16.05)	10.50 (8.90–16.10)
**Ai** (cm/s)	5.20 (4.0–7.30)	5.85 (4.98–7.58)	6.70 (4.90–7.48)**	4.40 (3.65–4.75)**	5.40 (4.80–7.80)
**St** (cm/s)	9.20 (7.60–13.90)	7.90 (6.73–10.10)	9.10 (6.65–11.95)	6.60 (6.05–9.0)	11.10 (7.85–11.45)
**Et** (cm/s)	10.20 (8.30–11.80)	8.85 (8.38–10.75)	9.25 (8.05–11.5)	7.60 (7.10–9.55)	10.30 (9.25–11.5)
**TVE/Et** (cm/s)	7.38 (5.83–8.27)	6.17 (4.38–7.24)	7.03 (5.60–8.09)	5.26 (4.23–8.91)	7.44 (5.93–8.52)
**At** (cm/s)	7.60 (7.10–11.90)	8.70 (6.33–10.78)	7.75 (5.70–8.50)**	5.10 (4.60–6.75)**	8.30 (6.55–9.95)
**GSAplax** (%)	19.30 (14.56–23.08)	18.70 (16.75–21.20)	18.80 (13.68–21.4)	15.80 (13.80–20.35)	20.35 (16.90–26.53)
**GSLV4ch** (%)	20.05 (16.68–21.05)	19.30 (14.50–21.90)	16.40 (12.75–20.9)	17.50 (13.95–19.60)	20.60 (17.33–22.03)
**GSLV2ch** (%)	21.20 (16.33–23.0)	19.10 (14.75–21.30)	17.90 (12.50–18.95)	13.70 (11.90–18.75)	21.50 (16.2–28.48)
**GSLV** (%)	19.60 (18.35–22.03)	18.83 (16.60–21.85)	18.82 (12.68–21.10)	15.67 (13.75–19.0)	21.90 (17.25–24.2)
**GSRV** (%)	21.05 (16.10–24.08)	17.15 (14.28–19.73)	18.1 (14.80–22.93)	14.40 (12.20–16.15)	21.80 (19.4–25.29)

Data are presented as medians (interquartile range: 25th – 75th percentile), *Ai* Late diastolic interventricular annular velocity, *Am* Late diastolic mitral annular velocity, *At* Late diastolic tricuspid annular velocity, *BOAS* Brachycephalic obstructive airway syndrome, *BST* Boston Terriers, *Ei* Early diastolic interventricular annular velocity, *Em* Early diastolic mitral annular velocity, *Et* Early diastolic tricuspid annular velocity, *FB* French Bulldogs, *GSAplax* Left ventricular global strain in apical long axis 5 chamber view, *GSLV* Mean left ventricular global strain, *GSLV4ch* Left ventricular global strain in apical long axis 4 chamber view, *GSLV2ch* Left ventricular global strain in apical long axis 2 chamber view, *GSRV* Right ventricular global strain in apical long axis view, *MVA* Late diastolic mitral wave, *MVE* Early diastolic mitral wave, *MVE/A* early diastolic mitral wave to late diastolic mitral wave ratio, *Si* Peak systolic interventricular annular velocity, *Sm* Peak systolic mitral annular velocity, *St* Peak systolic tricuspid annular velocity, *TVA* Late diastolic tricuspid wave, *TVE* Early diastolic tricuspid wave

✩ = significant differences between brachycephalic dogs with signs of BOAS and non-brachycephalic dogs (data presented in Tables 2 and 3), ** = significant differences between brachycephalic dog with and without signs of BOAS

#### Boston terries with signs of BOAS compared to non-brachycephalic dogs

Boston Terriers with signs of BOAS had larger CVCII (*p* = 0.001) and CVCEI (*p* = 0.006), higher FS (*p* = 0.008), and lower CVCCI (*p* = 0.004) and Si (0.047) than non-brachycephalic dogs (Tables 4 and 5).

#### Pugs with signs of BOAS compared to non-brachycephalic dogs

Pugs with signs of BOAS had smaller LALaxI (*p* = 0.001), RVAdI (*p* = 0.019), and RVAsI (*p* = 0.034) and lower MAPSEI (*p* = 0.019), Am (*p* = 0.014), and Si (*p* = 0.008) than non-brachycephalic dogs (Tables 4 and 5).

### Brachycephalic dogs with signs of BOAS compared to brachycephalic dogs without signs of BOAS

#### French Bulldogs with signs of BOAS compared to French Bulldogs without signs of BOAS

French Bulldogs with signs of BOAS had smaller LALaxI (*p* = 0.014), larger IVSsI (*p* = 0.012), lower early diastolic mitral wave velocity to early diastolic tricuspid wave velocity (MVE/TVE) ratio (*p* = 0.001), and higher tricuspid early diastolic wave velocity (TVE) (*p* = 0.005) than FB without signs of BOAS (Tables 4 and 5).

#### Pugs with signs of BOAS compared to Pugs without signs of BOAS

Pugs with signs of BOAS had higher TAPSEI (*p* = 0.020), Ai (*p* = 0.014), and late diastolic annular velocity of the right ventricular free wall (At) (*p* = 0.049) and lower MVE/Em (*p* = 0.049) than asymptomatic P without signs of BOAS (Tables 4 and 5).

## Discussion

The results of this study showed significant differences in echocardiographic parameters between the dogs of the three brachycephalic breeds and non-brachycephalic dogs. In addition, there were significant differences in echocardiographic parameters between dogs with and without signs of BOAS.

Brachycephalic dogs (with and without signs of BOAS) had significantly higher LA/Ao and MVE/Ei ratios, a significantly smaller LVIDdI, and significantly lower TAPSEI, Am, Si, Ai, and GSRV than non-brachycephalic dogs. A higher LA/Ao ratio was previously reported in brachycephalic dogs [33, 36–38] and in patients with OSA [39–44]. In Boxers [36] and English Bulldogs [37], the larger LA/Ao ratio is explained by the breed-specific smaller Ao. There is no consensus on LA dilatation in OSA patients [45]. Some authors reported that the structural and functional remodeling of the left atrium is proportional to the severity of OSA [39–44, 46], while others found no difference between the different stages [47, 48]. The significantly higher MVE/Ei ratio in brachycephalic dogs in our study may indicate higher left atrial filling pressure. Several studies have shown that the MVE/Em ratio is higher in patients with OSA [49–54]. In patients with OSA, the cardiovascular system is exposed to cycles of noxious agents such as hypoxia, hypercapnia, excessive negative intrathoracic pressure, and frequent awakenings from sleep due to repeated transient pauses in breathing, which impairs myocardial systolic function, activation of the sympathetic nervous system, and suppression of parasympathetic activity, thereby increasing heart rate, blood pressure, and myocardial wall stress, provoking oxidative stress and systemic inflammation, activating platelets, and impairing vascular endothelial function [20, 26].

The significantly lower TAPSEI and significantly decreased GSRV in brachycephalic dogs in our study could both describe decreased RV systolic function. In human patients, the effect of OSA on RV strain values is controversial [55]: In some studies, lower RV strains are associated with OSA severity [54, 56] whereas in other studies, no significant correlation between strain values and OSA was found [56, 57]. Unlike tissue Doppler imaging, speckle tracking echocardiography is not angle dependent and therefore provides a more accurate description of global and segmental systolic function [57, 55]. In patients with severe OSA, GSLV is significantly decreased compared with the control group [49]. Similarly, a decrease in Si in brachycephalic dogs in our study could represent decreased systolic motion of the interventricular septum in brachycephalic dogs. Significantly lower Am and Ai in brachycephalic dogs compared with control dogs could represent decreased LV diastolic function. Mean Am velocities were higher in patients with severe OSA than in control subjects [43].

In our study, FB had a significantly larger LA/Ao ratio and significantly lower TAPSEI, Si, Am and GSRV than non-brachycephalic dogs. In addition, FB had significantly higher MVE and significantly lower CVCCI compared with non-brachycephalic dogs. Higher MVE could indicate increased left atrial pressures and/or diastolic dysfunction. However, in patients with OSA, MVE [41] and MVE/A [49–51, 58] decreased with the severity of OSA. Significantly lower CVCCI in FB compared with non-brachycephalic dogs indicates higher right atrial pressures.

Compared with non-brachycephalic dogs, P had smaller LVIDdI and lower TAPSEI, Am, and Si. In addition, compared to non-brachycephalic dogs, P had smaller LALaxI, RVAdI, and RVAsI; lower MAPSEI, MVA, Sm, Em, Ei, TVE/Et, and mean GSLV; and higher MVE/Em. Smaller RVAdI and RVAsI might be related to breed and emphasizes that breed-specific echocardiographic reference values should be used in clinical practice. Lower MAPSEI, TAPSEI, mean GSLV, Si, and Sm may indicate decreased systolic function in P. Lower TAPSE has been reported in P compared with values of generic interbreed references [34]. Significantly lower Ei, Em, and Am might indicate decreased LV diastolic function. Higher TVE/Et and MVE/Em ratios may signify higher ventricular filling pressures and ventricular diastolic dysfunction. The MVE/Em ratio is higher in patients with OSA [49–54].

In this study, BST had larger LA/Ao and lower Si compared with non-brachycephalic dogs. In addition, compared with non-brachycephalic dogs, BST had higher CVCII and CVCEI, higher FS, and lower CVCCI. Higher CVCII and CVCEI could be breed-specific or due to higher intracardiac and intrathoracic pressure, whereas lower CVCCI indicates higher right atrial pressures. Higher FS and smaller LVIDs were previously found in FB, and the authors hypothesized that this may indicate increased mechanical activity of LV in brachycephalic dogs due to stress and activation of the sympathetic nervous system [33].

French Bulldogs with signs of BOAS had smaller LALaxI and RVAsI, higher FS, higher CVCI, and lower CVCCI, Am, and Si than dogs in the non-brachycephalic group. A smaller LA in FB with signs of BOAS than in non-brachycephalic dogs might be related to right-sided pressure and volume overload, but the result is controversial. There is no consensus on LA dilatation in OSA patients [45]; in some studies, left atrial remodeling is proportional to the severity of OSA [39–44, 46], whereas in others, there is no difference between the different stages of OSA [47, 48]. Lower CVCCI is consistent with higher right atrial pressures. The enlarged intrathoracic pressure fluctuations are due to obstructive airways, which increases venous return and contributes to RV volume overload [18]. In the late stage of OSA, pulmonary hypertension leads to RV pressure overload as a result of repetitive nocturnal hypercapnia and arterial hypoxemia [18]. Under hypoxic conditions, the pulmonary artery constricts primarily due to the ability of its smooth muscle to detect changes in the partial pressure of oxygen (PaO_2_). An increase in pulmonary vascular resistance exerts pressure overload on the RV, leading to hypertrophy with subsequent dilatation [18, 19]. A low Si may indicate decreased systolic function of the interventricular septum, and a low Am could indicate decreased LV diastolic function.

Boston Terriers with signs of BOAS had higher CVCII and CVCEI, higher FS, and lower CVCCI and Si than non-brachycephalic dogs. The lower CVCCI in dogs with signs of BOAS suggests higher diastolic pressures in the right heart. Higher FS is likely consistent with increased mechanical activity of LV due to stress and an activated sympathetic nervous system [33]. A lower Si in BST with signs of BOAS than in non-brachycephalic dogs may indicate a lower systolic function of the interventricular septum or a change in the interplay between the left and right heart due to changes in intracardiac and intrapleural pressures.

Pugs with signs of BOAS had smaller LALaxI, RVAdI, and RVAsI and lower MAPSEI, Am, and Si than non-brachycephalic dogs. A smaller LALaxI in P with signs BOAS than in non-brachycephalic dogs is a finding consistent with that of FB with BOAS compared with non-brachycephalic dogs. Higher intrathoracic pressures in brachycephalic dogs and OSA patients should increase myocardial wall stress. Obstructive sleep apnea is associated with LV hypertrophy even in the absence of hypertension, obesity, and diabetes [41, 56, 59]. A low MAPSEI likely reflects decreased LV systolic and diastolic function. A lower Si is consistent with decreased systolic function of the interventricular septum, and a lower Am might indicate decreased ventricular diastolic function. A smaller RVAsI in P with signs of BOAS than in non-brachycephalic dogs might indicate better RV systolic function; however, the result is controversial. Patients with moderate to severe OSA have higher indexed RV end-diastolic and end-systolic volumes compared with control subjects [43, 47]. In a study of 22 FB and 6 Beagle control dogs, RV changes were not statistically significant, although right ventricular remodeling was expected, and the authors hypothesized that it would become more evident with age when the condition becomes more chronic [33]. In addition, authors have speculated that more sensitive techniques such as MRI [60] may be needed to document early morpho-functional changes in the RV [33].

When comparing FB with and without signs of BOAS, FB with signs of BOAS had a lower MVE/TVE ratio and higher TVE than FB without signs of BOAS. A lower MVE/TVE ratio and higher TVE could indicate higher RA pressures in FB with signs of BOAS.

Pugs with signs of BOAS had higher TAPSEI, Ai, and At and lower E/Em than P without signs of BOAS. Higher TAPSEI could indicate better RV systolic function; however, this result is controversial because patients with OSA [50, 56], dogs with pulmonary hypertension [61], and P [34] were found to have lower TAPSEI than controls. In a study investigating the effects of increasing severity of BOAS in 42 P [34], other echocardiographic parameters, except for peak pulmonic velocity, showed no association with BOAS. However, tissue Doppler and strain parameters were not examined in the latter study. Higher Ai and At might indicate changes in right ventricular diastolic function, whereas a lower E/Em ratio implies lower left ventricular diastolic pressures. This result contrasts with findings in OSA patients, in whom a higher MVE/Em ratio was found [49–54]. The most likely reason for this finding is the small number of brachycephalic dogs without signs of BOAS. In addition, we hypothesize that dogs in the group without signs of BOAS may also have changes compared to BOAS due to their brachycephalic anatomy, which could lead to some ambiguous results. The small number of dogs without signs of BOAS and their classification as dogs without signs of BOAS without exercise testing and/or endoscopic examination of upper airways is another limitation of this study and the reason why dogs without signs of BOAS were not compared with non-brachycephalic dogs in this study. It should be noted that transthoracic echocardiography, the primary tool for assessing cardiac structure and function in dogs, is challenging in brachycephalic dogs because of dorsoventral compression of the thorax, obesity, and narrow intercostal spaces [62]. In addition, in brachycephalic dogs with signs of BOAS, quiet breathing is often not possible during echocardiographic examination because many dogs with signs of BOAS had difficulties ventilating quietly and lying on their sides. A limitation of this study is also the inclusion of dogs with different stages of BOAS.

Overall, the observed differences in echocardiographic parameters between brachycephalic and non-brachycephalic dogs suggest higher filling pressures in the right and left atria and some degree of diastolic dysfunction of both ventricles. These differences became even more apparent when brachycephalic dogs with signs of BOAS were compared with non-brachycephalic dogs and when brachycephalic dogs with signs of BOAS were compared with brachycephalic dogs without signs of BOAS. Higher diastolic pressures in the right heart could lead to cardiac remodeling; however, in our study, the right atrium and right ventricle were not larger in brachycephalic dogs and dogs with signs of BOAS. The most likely reason for this finding is the young age of the dogs included in our study; the progression of BOAS and cardiac remodeling are age-dependent, it takes time for the severity of anatomical changes to affect the heart, and therefore the secondary cardiovascular changes observed in our study are mild.

## Conclusions

We found significant differences in echocardiographic parameters between dogs of the three brachycephalic breeds and non-brachycephalic dogs, implying that breed-specific echocardiographic reference values should be used in clinical practice. In addition, significant differences were observed between brachycephalic dogs with and without signs of BOAS. The observed echocardiographic differences suggest higher right heart diastolic pressures affecting right heart function in brachycephalic dogs with and without signs of BOAS, and several of the differences are consistent with findings in OSA patients. Most of the changes of the heart morphology and function can be attributed to brachycephaly alone and not to the symptomatic stage.

## Methods

### Dogs

We included client-owned brachycephalic dogs of three breeds: FB, P, and BST. Dogs without signs of BOAS were diagnosed based on the absence of clinical signs (stertor, stridor, exercise, heat intolerance at rest and during daily activities, including running) and visible abnormalities such as stenotic nares. Dogs with signs of BOAS were classified based on clinical signs of exercise intolerance and other signs of BOAS. In dogs with signs of BOAS, abnormalities consistent with BOAS were confirmed under anesthesia by endoscopy by an experienced veterinary surgeon (VE) and the disease was graded as described previously [35, 62]. The control group consisted of healthy non-brachycephalic dogs with normal cardiac auscultation invited to participate in the study. All owners signed an informed consent form to participate in the study.

### Echocardiography

According to the guidelines [63–66], a complete echocardiographic examination of the left and right heart was performed by two experienced veterinarians (ADP, MB). A Vivid E9 (GE Healthcare, Europe) echocardiography system and a 1.75–3.5 or 4–10 MHz transducer were used. An ECG limb lead II was recorded simultaneously. Loops and images were analyzed using an offline Echo-PAC workstation (GE Healthcare, Europe). The echocardiographic examinations were performed in conscious dogs, with quiet breathing when possible. All echocardiographic parameters were measured for three to five heartbeats.

The following echocardiographic measurements were performed:

**- Two-dimensional:**
  From the right parasternal long and short-axis view: diameter of the left atrium in long (LALax) (cm) and short axis (LASax) (cm), and aorta (Ao) (cm) and LA/Ao ratio.
  From the left apical four-chamber view focused on the right ventricle: right ventricular internal diameter just below the tricuspid annulus (RVIDbas) (cm), right ventricular internal diameter in mid cavity (RVIDmid) (cm), right ventricular longitudinal diameter (RVIDlong) (cm) from the line of the tricuspid annulus to the inner edge of the RV apex, right ventricular area in diastole (RVAd) (cm^2^), right ventricular area in systole (RVAs) (cm^2^), fractional area change of the right ventricle (FAC) (%), and right atrial area (RAA) (cm^2^).
**- M-mode:**
  From the right parasternal short-axis view: thickness of the interventricular septum in diastole (IVSd) (cm) and in systole (IVSs) (cm), left ventricular internal diameter in diastole (LVIDd) (cm) and in systole (LVIDs) (cm), thickness of the left ventricular posterior wall in diastole (LVPWd) (cm) and in systole (LVPWs) (cm), and fractional shortening of the left ventricle (FS) (%).
  From the left parasternal apical 4-chamber view: mitral and tricuspid annular plane systolic excursion (MAPSE and TAPSE, respectively) (cm).
  From the left parasternal cranial view: vena cava at expiration (CVCE) (cm) and inspiration (CVCI) (cm). Caudal vena cava collapsibility index (CVCCI) was calculated as (CVCE–CVCI)/CVCE*100% (%).
**- Spectral and color flow Doppler velocities:** MVE and MVA (m/s), early diastolic mitral wave velocity to late diastolic mitral wave velocity ratio (MVE/MVA), TVE (m/s) and late diastolic tricuspid wave velocity (TVA) (m/s), and early diastolic tricuspid wave velocity to late diastolic tricuspid wave velocity ratio (TVE/A). The following ratios were also calculated: MVE/TVE and MVA/TVA.
**- Tissue Doppler annular velocities:** Sm, Em, Am, Si, Ei, Ai, and peak systolic and early and late diastolic annular velocity of the right ventricular free wall (St, Et, and At, respectively) (cm/s). The following ratios were also calculated: MVE/Em, MVE/Ei, and TVE/Et.
**- Two-dimensional speckle tracking echocardiography:** the global longitudinal strain of the left and right ventricle (%), using GE Echopac software. Left ventricular global strain (GSLV) was averaged using three views: left apical long axis 5 chamber view (GSAplax), left apical long axis 4 chamber view (GSLV4ch), and left apical long axis 2 chamber view (GSLV2ch). Right ventricular global strain (GSRV) was measured in the left apical 4-chamber long axis view focused on the right ventricle.

Weight-dependent parameters were indexed: one dimensional parameter as parameter/weight^1/3^ and two-dimensional parameters as parameter/weight^2/3^ [67].

### Statistical analysis

Data were analyzed with commercial software (SPSS, IBM SPSS 24.0, Chicago, IL, USA). The Shapiro–Wilk test was performed to test the distribution of the data. Because most of the data were not normally distributed, the Mann–Whitney test was used to test for statistically significant differences in age, weight, and echocardiographic parameters between the group of all brachycephalic breeds and non-brachycephalic dogs and between individual brachycephalic breeds (P, FB, BST) and the group of non-brachycephalic dogs. The Mann–Whitney test was also used to compare all data between brachycephalic dogs with signs of BOAS and non-brachycephalic dogs and between FB and P with and without signs of BOAS. The latter was not possible in BST because there were only three dogs without signs of BOAS of this breed.

Results are expressed as median and interquartile ranges (IQR; 25th to 75th percentiles). A value of *p* < 0.05 was considered significant.

## Data Availability

All data are available from the corresponding author on reasonable request.

## Abbreviations

Ai: Late diastolic annular velocity of the interventricular septum
Am: Late diastolic annular velocity of the left ventricular free wall
Ao: Aorta in short axis
AoI: Aorta in short axis index
At: Late diastolic annular velocity of the right ventricular free wall
BOAS: Brachycephalic obstructive airway syndrome
BST: Boston terrier
CVCE: Caudal vena cava at expiration
CVCEI: Caudal vena cava at expiration index
CVCI: Caudal vena cava at inspiration
CVCII: Caudal vena cava at inspiration index
CVCCI: Caudal vena cava collapsibility index
Ei: Early diastolic annular velocity of the interventricular septum
Em: Early diastolic annular velocity of the left ventricular free wall
Et: Early diastolic annular velocity of the right ventricular free wall
FAC: Fractional area change of the right ventricle
FB: French Bulldog
FS: Fractional shortening of the left ventricle
GSAplax: Left ventricular global strain in apical long axis 5 chamber view
GSLV4ch: Left ventricular global strain in apical long axis 4 chamber view
GSLV2ch: Left ventricular global strain in apical long axis 2 chamber view
GSRV: Right ventricular global strain in apical long axis view
IVSd: Thickness of the interventricular septum in diastole
IVSdI: Thickness of the interventricular septum in diastole index
IVSs: Thickness of the interventricular septum in systole
IVSsI: Thickness of the interventricular septum in systole index
LALax: Diameter of the left atrium in long axis
LALaxI: Diameter of the left atrium in long axis index
LASax: Diameter of the left atrium in short axis
LASaxI: Diameter of the left atrium in short axis index
LA/Ao: Ratio of the left atrium to aorta in short axis
LVIDd: Left ventricular internal diameter in diastole
LVIDdI: Left ventricular internal diameter in diastole index
LVIDs: Left ventricular internal diameter in systole
LVIDsI: Left ventricular internal diameter in systole index
LVPWd: Thickness of the left ventricular posterior wall in diastole
LVPWdI: Thickness of the left ventricular posterior wall in diastole index
LVPWs: Thickness of the left ventricular posterior wall in systole
LVPWsI: Thickness of the left ventricular posterior wall in systole index
LV: Left ventricle
MAPSE: Mitral annular plane systolic excursion
MAPSEI: Mitral annular plane systolic excursion index
MVA: Late diastolic mitral wave velocity
MVE: Early diastolic mitral wave velocity
MVE/A: Early to late diastolic mitral wave ratio
OSA: Obstructive sleep apnea
P: Pug
RAA: Right atrial area
RAAI: Right atrial area index
RV: Right ventricle
RVAd: Right ventricular area in diastole
RVAdI: Right ventricular area in diastole index
RVAs: Right ventricular area in systole
RVAsI: Right ventricular area in systole index
RVIDbas: Right ventricular internal diameter just below the tricuspid annulus
RVIDbasI: Right ventricular internal diameter just below the tricuspid annulus index
RVIDmid: Right ventricular internal diameter in mid cavity
RVIDmidI: Right ventricular internal diameter in mid cavity index
RVIDlong: Right ventricular longitudinal diameter
RVIDlongI: Right ventricular longitudinal diameter index
Si: Peak systolic annular velocity of the interventricular septum
Sm: Peak systolic annular velocity of the left ventricular free wall
St: Peak systolic annular velocity of the right ventricular free wall
TAPSE: Tricuspid annular plane systolic excursion
TAPSEI: Tricuspid annular plane systolic excursion index
TR: Tricuspid regurgitation
TVE: Early diastolic tricuspid wave velocity
TVA: Late diastolic tricuspid wave velocity
TVE/A: Early to late diastolic tricuspid wave ratio
